# 

**Published:** 1859-08

**Authors:** 


No. III. AUGUST—1859. Vol. XV.

### NEW YORK MONTHLY REVIEW

OF

### Medical & Surgical Science,

AND

### BUFFALO MEDICAL JOURNAL.

EDITED BY AUSTIN FLINT, Jr., M. D.

Prof. of Physiology and Microscopic Anatomy in the New York Medical College.

NEW YORK:

CHARLES B. NORTON, APPLETON'S BUILDING,

346 AND 348 BROADWAY.

BUFFALO:

A. I. MATHEWS, No. 220 MAIN STREET.

*LONDON:* H BAILLIÈRE, 219 REGENT STREET. *PARIS:* J. B. BAILLIÈRE ET FILS, RUE HAUTEFEUILLE. *MADRID:* C. BAILLY-BAILLIÈRE, CALLE DEL PRINCIPE. *LEIPZIG:* BERNARD HERMANN, AGENT OF B. WESTERMANN & CO.

Price $2.50, payable in advance, or $3.00 if not paid till the end of the year.

